# Mercury and the Liver

**Published:** 1893-05

**Authors:** A. Bethune Patterson

**Affiliations:** Atlanta, Ga.; Late Assistant to the Royal London Ophthalmic Hospital, and Golden Square Throat Hospital, London, England; 239 Equitable Building


### MERCURY AND THE LIVER.

BY A. BETHUNE PATTERSON, M. D., ATLANTA, GA.

Late Assistant to the Royal London Ophthalmic Hospital, and Golden Square Throat Hospital, London, England.

The claim that Mercury has as a therapeutic agency upon clinitions dates back to the primitive periods of the healing art, ages before the most enthusiastic supporters of medicine would have ventured the prediction of its present position among the honored branches of science. As a remedy Mercury has passed through many vicissitudes, fluctuating in popularity, the outcome and the result of an empirical and injudicious administration, such an experience was calculated to bring reproach upon a remedy pregnant with great force and activity. The evils that sprang up from such a course engendered a spirit of distrust which naturally ripened into prejudice, threatening to exclude it from the list of remedies. The swaying of public caprice and professional disfavor could not assign a remedy so potent for good, to oblivion. The development of scientific research has placed Mercury at the head of the list of remedies, and with an understanding of its modus operandi, its field of usefulness will widen, and the evils which grew out of the empirical application will lessen. To intelligently observe and comprehend the therapeutic effects of a remedy, the physiological, pathological and etiological laws must be considered, a brief survey of these fields of study, and my interpretation of the laws governing them, have led me to the theory here advanced, as to the controling influence of Mercury on the pathological conditions of the alimentary tract and liver. It is through the portal system that the elements that go to make bile are transported. This system of vessels contain all the blood gathered from the alimentary tract together with the new elements of the digestive action save that which is taken to the circulation by the chylous vessels. The portal vein on entering the gland splits up into small branches, interlobular veins, thence into the net-work of blood capillaries, it is upon these capillaries that rest the hepatic cells in which is elaborated the liquid bile; these cells are further to be regarded as sentinels guarding the portals of the circulation against the invasion of extraneous matter from the alimentary tract.

In making a microscopical dissection of the tissues of the liver and other organs, we are brought to consider an element in their construction which claims special attention, I alude to the cells of the epithelial variety, they are described as being made up of protoplasm nucleus, nuclei, matrix and intra-nuclear network with its nucleoli. The laws which govern cells and their complex structure, are not unlike other forces in nature—a hidden mistery—we know of their presence by the evolutions and revolutions that are ever taking place. Thus veiled in from the scrutiny of the scientist they can only be considered by persuing the rules governing deductive reasoning. What are these forces? A similarity is to be found in the anatomical construction of cells while their action and function has a broad latitude. Compare surface cells internal and external with those found in the structure of glands, those secreting the saliva an alkaline fluid holding a digestive ferment with those lining the gastric tubules and surface of the stomach, eliminating gastric juice along with its characteristic ferment. The observations upon the white corpuscle (amœbic cells) the active and intelligent part performed in certain pathological conditions throws much light upon cell function, we are led to regard micro-organism as simple forms of cell life, their propagation by division as observed in some forms, their reaction to staining solutions demonstrating conclusively the presence of nuclei. Modern research has established the fact that the conditions spoken of as fermentation has its origin in the germination of micro-organisms. Thus it is growing to be the custom to classify disease producing germs under the head of ferments.

The characteristic phenomena that attend the propagation of this class of cells are so distinctive that in instances where such phenomena appear and cannot be traced to special forms of micro-organism, it goes without saying that it would be a logical conclusion to claim their presence. The progress in this line of study justifies the conclusion that, the physiological differences observed in cell action is due to hypothetical forms of micro-organisms, possessing distinctive anatomical physiological and biological points of differences, as are observed in bacterium aceti, bacterium of lactic and butyric acid, and many others, the morphology of these bacteria are clearly understood, with improved microscopes and methods of cultivating, isolating and staining bacteria. We predict the time is not far distant when scientists will demonstrate the existance in the cells of the stomach of a bacterium which produces the hydrocloric acid and peptone, a liver constantly presides over the glycocholic and other functions. The powers that lie in the cells to digest assimulate and appropiate the elements of nutrition are identical with those constantly operating to dispose of extraneous and disease producing elements. In speculating upon the departures of a healthy or physiological action of the liver and alimentary tract, a study of the physiological and etiological factors and laws governing the operation of the latter upon the former will aid and assist the therapeutist in an intelligent and scientific application of remedies. In looking out for the causes producing the aforesaid disturbances, our investigations at once center upon micro-organisms, the source of fermentation. Bacteria and their germs are being systematically introduced into the alimentary tract in company with food and drink, and the failure of the digestive forces to dispose of them, insures formations of lively colonies, which in turn act as a disturbing influence upon the correllation of the physiological functions. The elements of nutrition in company with micro-organisms, germs and ptomaines are absorbed alike, and in course of their pilgrimage to the general circulation, a large percentage take passage through the portal system: when they have gained entrance to the cells of the liver and the other tissues of the body, the cell forces are at once set in operation looking to the destruction and elimination of the extraneous matter. Thus the physiological energies are diverted, and we at once experience the constitutional disturbances peculiar to the various pathological conditions established. It is in keeping with observations in other fields to expect retarded physiological action—when the energies are being expended in defensive action. Thus it is, a general septic condition prevails attended with its characteristic symptoms—lassitude, headache, nausea and the so-called bilious attack.

In selecting remedies to meet the above indications, we are brought to consider that class of agents that come under the head of anti-ferments and antiseptics, remedies that will hold in check the germination and development of germs, as such are readily disposed of by the cells and are powerless to effect any changes in the tissues. It is only after they have germinated into microbes that pathological conditions are established. Such a remedy possessing a cathartic action becomes the ideal one. Mercury meets the requirements, and if its usefulness can be explained at all it is upon the lines above set forth. The presence of Mercury in the stomach and liver is sufficient to paralyze germ life without materially affecting the activity of the cells in their digestive and eliminative operations. The least quantity of Mercury that experience has demonstrated to meet the demands, should be adhered to, as quantities sufficient to kill cells, setting up rapid exfoliation would be reprehensible. The above effects are daily observed by those who make use of weak solutions of corrosive sublimate in surgical operations. The exfoliation of the stratified epithelium of the outer layer of the epidermis of the hands when allowed to remain in the solution for a short time to render them aseptic. A similar exfoliation would attend the application of mercury to cells internal, if in sufficient strength, thus ptyalism offers proof of this effect, the exfoliation of cells in the salivary glands goes on so rapidly that some time elapses before the cells regain sufficient force to protect the neighboring tissues from being made the germinating grounds of colonies of pyogenic germs that swarm in from the buccal cavity, after the exit of the Mercury. The germicidal properties of Mercury and other similar agencies are more thoroughly studied in mediums containing colonies of pure culture of bacteria, thus by this practical application and study we have gained very exact knowledge of this class of anti-ferments. The beneficial effects that accrue from a judicious administration of Mercury have been attributed to various imaginary influences, it heads the list of the so-called cholagogues. That famous report of the Edinborough Committee that was received and so generally criticised about twenty-five years ago, claimed that calomel possessed no specific action upon the liver. This expression proved very misleading to many at the time, and especially to those who were antagonistic to the use of the remedy, it was made use of to bolster up their objection. Notwithstanding the opposition to Mercury at that time, it now has few opponents among members of the profession that have kept pace with the progress of scientific medicine, and so it stands to-day one of a very few scientific remedies. As an antiseptic to the alimentary tract and liver it has no equal, holding at bay the fermentation processes, while its catharsis clears the system of the offending matter.

239 Equitable Building.

### FEVER.

BY L. B. ANDERSON, M. D., NORFOLK, VA.

As the disease advances, the above symptoms become more demonstrative, but more irregularly so. Delirium, sometimes wild, most generally low and muttering, persists during the evenings and nights—the eyes are either heavy and dim, or bright and sparkling—there is great wakefulness or heavy drowsiness, amounting to stupor. The hands and feet in the early morning are often cool, while the chest and abdomen are burning hot. The discharges all indicate a progressive tendency to putridity, and the evolution of ptomaines, which not only poison the subject, but contaminate the surrounding atmosphere, and are often so potent as to engender like contaminations, disorders, and diseases in others. It is now that the leucomainic accumulations cause those formidable and anomalous symptoms so often confronting the doctor in the progress of typhoid and other adynamic fevers — wild or muttering delirium, red, dry and fissured state of the tongue, contracted and sharp features, sleeplessness or stupor, constipation, with tympanitis, or diarrhœa with hæmorrhage, rapidly follow each other in their morbid train, so ominous, and where not prudently and philosophically met, so sure of dire results.

### TREATMENT.

In the space allotted us, we have tried to classify, arrange and systematize functional aberrations resulting in what is vulgarly known as *malarial* fever, so that each step of departure from health could be traced from the earliest departure in simple intermittent, to a fully developed typhoid fever. By the adoption of this arrangement, the principles of treatment can also be systematized and arranged, meeting the sanative indications as the morbid aberrations demand.

In giving the "treatment" under the head of Intermittent Fever, we only designed to indicate the principles by giving the simplest and briefest outline possible, in preparation for what was to follow.

The first demonstrative indications of an attack of fever are sensations of coldness generally along the spine, then the hands and feet, and soon, as previously stated, invading all the person. In an ordinary chill, the services of a physician are scarcely ever required, for hot applications to the feet and legs, and back, generally cause reaction in a short time. But it is sometimes the case, that when the centripetal tide begins its inflow, it continues until all the surface is as algid as a winter's marble, the skin is bathed in a cold and copious sweat, the breathing is hurried and short, the pulse is exceedingly feeble and frequent, when it can be felt at the wrist, but often it is not discernable in that locality—generally there is a sense of oppression accompanied with consuming heat at the præcordia, the countenance is generally calm and serene, bearing the impress of mental vacuity; the tide of life is ebbing fast and what can be done must be speedily and philosophically done, or all will be of no avail. What are the scientific indications?

The blood has accumulated in the vessels of the internal organs, all its heterogeneous constituents are promiscuously mingled, and as a necessary consequence the vital laws are suspended and chemical changes are rapidly effected, evolving volcanic heat, which seems to consume the living tissues. It is now that leucomaines are evolved, which speedily obtund the sensibilities, prostrate the nervous centres, increase the inflow of blood, leave all the cutaneous vessels and nerves insensible to any ordinary impression, of heat, stimulants, sinapisms or epispastics. Internal stimuli only increase the fires within and add fuel to the flame, and what then can be done? Extinguish the internal heat, drive the blood from the overloaded veins and arteries by arousing their functional activity, and arouse the sensibility of the nerves and capillaries on the surface, by an agent which, as soon as applied to a living tissue bathed in sweat, excites, almost instantaneously, such intense burning as to cause the almost moribund subject to cry out with pain. The first indication is promptly and almost infallibly met by the free use of crushed ice, the second by the application of chloroform. Give the pounded ice in tablespoonful portions, urging the patient to swallow it so soon as it enters the mouth, and repeating it as rapidly as possible till reaction occurs. In the meantime, saturate a handkerchief, folded some four inches square, with chloroform and apply to the epigastrium. It will scarcely touch the surface before a cry of pain will be uttered. Then move it from place to place over the abdomen, chest and back. Generally, in ten or fifteen minutes, after the use of the ice is begun, the perspiration will dry up, the pulse will return at the wrist, the internal heat will cease, the carotid and temporal arteries will throb, the skin will become burning hot, and reaction will be established.

If the secretions are suspended and the bowels are torpid, give a mild cholagogue cathartic and cooling diuretics. Ordinarily an intermission will follow in one or more hours, when quinine in five grain doses should be given every hour, till fifteen grains are administered, and then every two hours till twenty-five grains have been given. This will generally secure the patient from another congestion. Two grain doses of quinine should be given thereafter every four hours for a day or two, and then every morning for several days; strict attention given, all the while, to all the functions and to the diet.

When called to a patient who has been complaining for several days, with headache, pain in the back and limbs, dry mouth, furred tongue, loose or constipated bowels, scanty and high colored urine, restless nights, anorexia, rise of fever during the day and only a moderate decline in the morning, a most thorough and careful scrutiny of all the organs should be made before a step is taken. If no evidence of inflammation can be discovered in any organ or tissue, the trouble may be attributed exclusively to functional depravity. If the tongue be coated, the breath emitting a foul feverish odor, a callous, oppressed sensation in the stomach, a constricted, full sensation in the sub-epigastric region, an hour or two after eating, with accumulation of gasses, and a disposition to eructate, torpor of the liver and pancreas, and obstruction of the duodenal glands with mucus, may confidently be diagnosed. Whatever other aberrations may exist, these are sufficient to demand the use of such agents as will remove these troubles. To excite the torpid liver and pancreas, remove the obstruction from the duodenal glands, and liberate the pent up secretions, to a man, of ordinary stamina, give at bed-time—

℞ Calomel ........................................ grs 12
Comp. Ex. Colocynth.............................. grs 18
M—Capsules three. S—Administer at 8 P. M.

This will begin to act about 2 o'clock A. M. and will give two or three large bilio — mucus discharges, affording the most gratifying relief. In some instances, nothing more will be required to establish a safe basis for convalescence. But if the functions of the kidneys, the mucous lining of the Bronchia, and the mucous coats of the hepatic tubes are coated with mucus, the fever may be only greatly reduced by the purge, at about 8 or 10 o'clock a marked coldness of the extremities will be perceived, followed by a most demonstrative rise of fever. The indications now, will be to remove the accumulations from the membranes mentioned, excite the action of the kidneys and skin and energize the functions of the liver, pancreas and intestinal glands. To these ends:

1. ℞ Calomel ........................................ gr. 6.
Bicarb soda........................................ gr. 12.
Nitrate potass..................................... gr. 12. M.
Make Pulv. 12, S.: give one every 2 hours.

2. ℞ F. E. Ipecac and Tr. Aconite, aa.................. ℈i

Spts. Eth. Nitric.................................. ℥i. M.

S. 30 drops every hour.

At 5 o'clock the next morning give:

3. ℞ Cream Tartar........................................ ʒij

Bicarb. Soda........................................ ʒss M.

S. Administer while effervescing, in half a glass of water.

If there be no serious congestion in any important organ, and no latent inflammation, upon the action of the above a perfect intermission may be predicted at 7 or 8 o'clock, when you will have reduced a remittent to an intermittent fever, which must be treated accordingly.

Should, however, an intermission not be secured and the fever only partialy remits, to be followed by another demonstrative rise, you may be sure of the existence of congestion or subacute inflammation in some organ or tissue, overpowering the nervous centres and arresting the secretions.

A minute and scrutinizing examination of all the organs should be made, beginning with the brain and ending with the bladder and rectum. If there be a dull, heavy sensation in the brain, less than an acute pain, with a feeling of vertigo, dull expression of the eyes with dilated pupils, we may be assured of more or less torpor of that organ. In proportion to the congestion will there be a corresponding lethargy of all the vital organs, and hence an impaired state of their functions. It is of the first importance, therefore, that this important, vital organ should be freed from its incumbus. For this purpose, if the pericranial tissues be hot and dry, cloths wrung out of cold water should be continuously applied to the scalp, epispastics applied to the extremities, upper and lower, and the abdomen and spine rubbed with stimulating liniments.

Should, however, with heat and dryness of the pericranial tissues, there be throbbing of the arteries, acute pain, intolerance of light or sound, with mental aberration, besides the applications above advised, leeches should be applied to the temples, dry cups to the neck and spine, ℞ No. 2, be given in larger and oftener repeated doses, cooling aperients, if needed, administered. Instead of cerebral developments, you may find a throat, laryngeal, or bronchial complication. When it will be necessary to use—

4. ℞ Ol Piper Menth.................................℥ss

Ol Terebinth.................................℥ss

Ol Olive.................................℥j. M.

S—Liniment. Apply externally to the throat and chest.

At same time use ℞ No. 2, and with each dose ten drops of Tr. Sanguinaria.

Should, however, the local irritation be found in the abdominal viscera, the greatest discrimination must be used, both as to diagnosis and treatment, for it is here that leucomaines are so freely generated, contaminating with their poisonous emanations all the organs and prostrating the vital powers. If there be gastric irritation, or indications of increased sensibility of the intestinal tissues, the blandest nourishment should be given in small and frequently repeated portions—mucilages as drinks. Ipecac and aconite, with minute portions of Deod Tr. Opii should be given frequently, and the bowels kept well opened with mild aperients, should the alvine evacuations be devoid of bile, small doses of calomel and soda, or cœrul mass and soda, guarded, if diarrhœal tendencies indicate it, with one or two grains of Pulv. Doveri at night, to be followed the next morning with castor oil and oil turpentine. At the same time hot fomentations should be applied every hour, over the whole abdomen. If the kidneys are peculiarly torpid, frictions of No. 4 Liniment over back and bowels, and a flannel pad applied every hour, and Acetate Potaso given in five grain doses in conjunction with No. 2.

Sometimes the fever will continue when there is no very marked local or functional aberration to be found anywhere. But, be you sure the functions are more or less impaired and the secretions to a greater or less degree perverted. To meet the generally impaired condition's demands, strive to remove as speedily and effectively as possible all acid or effete metabolic accumulations, stimulate the liver, excite the functions of the kidneys and skin, and administer suitable nutrients.

Despite all scientific treatment, the prostrated and perverted condition of the vital powers and functions are such when the physician is first called in, as to render it impossible to meet all the indications satisfactorily. The liver may be very much congested, the gall bladder infarcted with black bile, requiring active medication to remove the trouble, but the irritation already lighted up by the retained acrid secretions in the *prima via* renders such a step of extremely doubtful propriety. And yet it is sometimes the case, that a full dose of calomel, and two or three grains of Dover's powder given at night and the abdomen kept swathed in flannel cloths, wrung out of hot water, followed the next morning with a tablespoonful of castor oil and half a teaspoonful of oil turpentine will act like a charm, removing a quantity of black viscid bíle and tenacious mucus, and restoring the hepatic secretion. The symptoms are greatly mollified, by such a result, the febrile excitement reduced and the case greatly simplified. When from the conspicuous development of Typhoid symptoms such a step may seem too rash, still it is best to hasten mildly and gain the above results as speedily as possible, so as to prevent ulceration of Peyer's glands and its troublesome consequences.

5. To this end: ℞ Cœrul Mass..................... ʒss.

Pulv. Dovers.................... ℈ss.

Bicarb. Soda.................... ℈ss. M.

Make 6 pills, S. give one every 4 hours.

℞ No 2 to be used every hour. Liniment No. 4 to be freely applied to the abdomen every hour and covered with a hot mush poultice. The last pill to be followed with the previously designated dose of oil and turpentine in two hours after the last is given.

Should the impaired condition of the functions still persist so as to keep up an ardent fever, No. 2 should be persisted in, in forty drop doses, an effervescing beverage of twenty grains of Tartaric Acid and twenty-five grains of Bi Carb. Soda, administered every two hours, the liniment and poultices continuously persisted in, and the whole body and extremities bathed every four hours in

℞ Ol Olivœ and Ol Morrhuæ aa.................. ℥ss

Alcohol q s ad................................ ℥4 M

S. Used as directed.

Every night five grains Blue Mass, five grains Bi Carb. Soda and two grains Pulv. Dov: to be given one every other morning, oil and turpentine to be given.

Typhoid symptoms often arise in the progress of an improperly treated or neglected, simple continued, functional, or, vulgarly called, bilious or malarial fever, such as we have above described. But in certain constitutions of the atmosphere the vital functions are so insidiously undermined that when the vital balance is lost, it is found that all the indications of a well marked typhoid fever are present. The secretions have been so stealthily perverted and the *prima via* so loaded with them, that leucomainic emanations have pervaded and overpowered all the nervous centres and vital organs, and discord and danger reign supreme. At this early stage, ulceration of Peyers glands so characteristic of the later stages of Adynamic-Enteric, or typhoid fever, has not occurred, and hence, the sooner the vitiated secretions are removed, the functions restored to healthy action, the sooner the evolution of leucomaines will be arrested and their dire effects removed. Brother Æsculapius, trouble not yourself about germicides to destroy the hypothetical "typhoid bacillus," for God in His goodness has sent bacterial scavengers, "each after its kind," to consume the death-dealing leucomaines and ptomaines, which are so rapidly evolved from retained vitiated animal matters, in the seething intestinal laboratory, by chemical laws. If, therefore, you can capture whole bottles full of these heaven sent messengers of mercy, pour them into this seething caldron, that they may consume the organic alkaloids as fast as evolved, and then prevent their poisonous effects upon the already overpowered organs. While typhoid fever is unquestionably produced by the imbibition of leucomanic emanations from the discharges of persons laboring under the disease, it is often developed as above stated, in the most cleanly, refined and scrupulously careful families, in the most healthy localities. If health be the exercise and expression of the perfect harmony of all the vital functions, disease must be a perversion or suspension of one or more of these functions, its potency being measured by the extent of departure from a normal status. The prerogative of the physician consists, therefore, not in combatting hypothetical or utopian etiological factors, but in restoring suspended or perverted functions.

We are called to a case which has been so insidiously progressing as scarcely to attract notice, for several days. The subject is restless, tortured more or less with pains in various localities; his flesh is dry and hot, his pulse feeble and frequent, his tongue coated except about the edges and tip, which are dry and red, yet with but little thirst, giddiness, no appetite, fullness about the stomach and bowels, constipation, or thin, watery discharges, devoid of bilious matter, urine scanty and acrid, trembling of the tongue and hands when protruded or extended, overpowering drowsiness, but sleep affording no refreshment, and attended with disturbed dreams; features sharp and contracted, expression of a high nervous tension, or relaxed and stolid, expressive of total unconcern and indifference, and withal, a sense of great lassitude and prostration.

There have been no exhausting evacuations, and overpowering shock, to account for such suppression of vital energy and exhaustive prostration. The debility is, therefore, negative, rather than positive, but none the less real. The vital functions are suspended, the products of retrogressive metabolism have accumulated in organs and tissues overpowering the nervous centres, and, hence, suppressing that influence which preserves a healthy influence over the circulation, nutrition and secretion, whereby a healthful balance is preserved. Derangement and discord reign supreme. Blood destined for one organ or tissue is thrown in promiscuous confusion upon others, vital laws no longer controlling the functions, the discordant elements of the abnormally freighted blood pass under the influence of chemical laws, and organic alkaloids of various qualities are being evolved from this active laboratory, some liquid and acid, flowing along the intestinal canal, irritating, burning and scalding, exciting inflammation in the mucous lining and glands thereof, others gaseous, possessing the properties of various vegetable alkaloids, such as veratrine, hyosciamine, morphine, atropine, etc., which pass readily through the animal membranes, and contaminate and overpower all the vital energies. It is where these poisonous emanations pass from the sick, through air, water or food, into the systems of others, that similar morbid developments are produced therein, hence there is a possibility of contracting disease faom any one in whom leucomaines are being freely generated.

In such general pathological developments, where disease reigns supreme, the most prompt, but prudent therapeutical measures are demanded. As the liver, pancreas, duodenum and intestinal glands, are the most important emulgents of the animal economy and the most readily responsive to remedial agents, because the most accessible, the attention of the physician should be first directed to them. Hence such agents should be used as would not only excite the secretory functions of the organs named, but would empty the alimentary canal, the great receptacle of vitiated matters, as well as the laboratory in which poisonous leucomaines are evolved. To unload the liver and excite its functional activity, mercury in the form of calomel or blue mass, or a combination of these, experience has demonstrated to be the most effectual. Being slow in its action, and often rendered partially inoperative by the plastic mucus lining the mucous membranes, soda and compound extract of colocynth or jalap are the most suitable adjuvants, hence

℞ Hydr: Chlo: Mitis.......................... ℈ss
Bi Carb. Soda.................................gr. v
Comp. Ex. Colocynth or Jalap.............gr. xv M
S: Administer at bed-time—will be found most effective.

A full dose, even larger quantities than above named, will generally act more effectively and much less harshly than smaller doses, because they relax all the emunctories, cause the secretions to flow freely and bear away all irritating matters and leave nothing behind to fret and irritate the bowels.

While the cathartic is doing its work in the alimentary canal and on its associated organs, the kidneys should be aroused to active work, to bear away the redundant urea and salts which have accumulated in the blood as a result of suspended secretions. To this end the free use of ℞ No. 2 should be employed, while hot fomentations should be applied to the abdomen, and the whole surface frequently bathed with ol. olivæ and alcohol, one to three.

These indications being fulfilled, the after treatment must be conducted on the general principles already promulgated. There are, however, certain developments in the progress of typhoid fever which demand special attention. For instance, when aperients have been neglected and acrid accumulations are suffered to remain in the intestines, phlogosis of the mucous membrane occurs and the distended coats of the capillaries permit the blood to escape by exosmosis, or ulceration destroys the coats of the vessels and hemorrhage follows, filling the bowels with blood. If anodynes and astringents be now administered the peristaltic action of the bowels is arrested and the effused blood passes rapidly into decomposition evolving leucomainic gasses, distendinging the bowels, increasing the hemorrhage and contaminating the whole system.

The indications, therefore, are to unstring the weakened or ruptured vessels, and at the same time to remove as speedily as possible the effused blood. To these ends, Ol. Terebinth, one drachm, and castor oil, half an ounce, should be promptly given, to be repeated every two hours until the bowels are freely opened. Then thirty minims of turpentine with a drachm Ol. of Olivae should be given every four or six hours. As a general rule the fever passes away with the hemorrhage and its removal, and the use of oil and turpentine as above indicated will complete the cure.

For many years the writer has had under his care a large number of typhoid and adynamic continued fevers, and not a case of hemorrhage has occurred, in which a dose of oil and turpentine is administered every other morning, which is his invariable custom, for, by this procedure, vitiated secretions, acrid and narcotic ptomaines are removed, and many of the more formidable complications are averted.

If unusual tenderness of the abdominal viscera continue, after the judicious use of the aperients and diaphoretics already prescribed in continued fever, the most salutary and satisfactory results are obtained by the application of a large blister over the whole abdomen retained for eight or nine hours, and the blistered surface dressed for several days, every four or six hours with a thickened milk poultice.

Despite our efforts to remove vitiated matters, restore the secretions and depurate the blood, it is often the case that violent cerebral complications arise, indicated by persistent stupor or wild dilirium. In such a condition, in addition to the industrious and judicious use of alteratives, aperients, diaphoretics, fomentations, embrocations, etc., as above recommended, the drawing of a blister on the back of the neck and between the shoulders, will be followed by the most satisfactory results.

We have said nothing about the use of quinine, the coal oil derivatives and stimulants in typhoid fever, because we find no philosophical justification for their use, and an ample trial of the first and last, many years ago, demonstrated that they were not only illusory and useless but absolutely injurious and hazardous, while a scrutinizing observation of the use of the second class of agents alluded to, in the hands of others, has convinced me that they should be placed in the same category.

DIET.

Without discrimination, we absolutely reject all animal extracts and broths, and the thousand and one peptonoids, and nameless preparations with which the market is flooded as food, for fever patients. Dr. Beaumont demonstrated in the case of Alexis St. Martin, that the coats of the stomach during fever were dry and phlogosed, and any solid food, or animal broths would be revolved for hours in the stomach without any change, and finally work their way, undigested, into the the duodenum, to become a source of irritation and oppression therein. Of what avail is it to pour into the stomach so-called nutritious aliments when that organ is utterly unable to digest them? The use of such materials in Typhoid fever, only increases the dryness of the stomach and the redness of the tongue, exalts the temperature, increases the restlessness, furnishes material for leucomaines, and increases the debility. Milk, first and last, milk diluted, milk boiled, milk thickened, sweetmilk, buttermilk, best milk all the the time, is the diet for fever patients. In the early stages of fever, only hot-water-tea, made by pouring as much boiling water on a half a cup of good rich milk, slightly sweetened, of which, a jelly glass-full should be given every two hours, either hot or cold, as the taste demands, Dr. Beaumont says, when such food is introduced into the stomach of a fever patient, in small quantities, it is rapidly absorbed and disappears. Even such simple food as this should not be given in too large quantities, else it will be left unabsorbed, and will pass in a coagalated state into the bowels, to irritate instead of nourish. A tablespoonful of milk readily digested and absorbed, will be assimilated and afford more nutriment and strength, than a pound of animal matter, which can not be thus assimilated.

To a fever patient in its early stage a pint and a half of hot water tea will afford ample nourishment for a young or small person, especially if they be fat, a quart should not be exceeded in anyone, during twenty-four hours. If there be any tendency to acidity of the stomach and hence, coagulation of the milk, a tablespoonful of lime-water should be added to the diluted water; to each glass, should there be a loose state of the bowels, the milk should be boiled, and in the advanced stages, thickened with arrow-root or corn-starch, a teaspoonful of either to a pint of milk. Occasionally a patient will be found who refuses to take milk in any combination. When this is the case the lightest, most nutritious, and digestible substitute can be easily prepared, thus: Take a quart of fresh wheat-bran, add three quarts of water and boil down to two quarts; strain and add a little sugar, nutmeg, etc., and give a wine-glassful every two hours. This was found in an old French Cordex and has been used with great satisfaction for many years, especially if there be any dysenteric complications. In the advanced stages, when there is great emaciation and debility, instead using alcoholic stimulants especially if the temperature be high, a liniment composed of olive oil one part, and pure alcohol two parts, applied every two or three hours, to the whole person will be found to subserve the most satisfactory purposes. Indeed, where the temperature is exalted and the skin dry and harsh, this liniment will, after a few applications, not only amply stimulate the person, but will reduce the temperature and pulse almost to a normal standard.

When all congestions, inflammations and perverted functions are removed and restored, and convalescence established the greatest care in the use of food and drinks should be observed for many days, both as to quality and quantity. Nothing is more important at this stage than to observe the old maxim, "make haste slowly

